# Screening Tools in School‐Based Health Centers for Children With Asthma

**DOI:** 10.1111/josh.70033

**Published:** 2025-06-20

**Authors:** Vanessa F. Maier, Olivia Dhaliwal, Amanda Liu, Kim Foreman, Matthew Linick, Katie Feldman

**Affiliations:** ^1^ Department of Family Medicine Case Western Reserve University School of Medicine Cleveland Ohio USA; ^2^ Department of Family Medicine The MetroHealth Medical Center Cleveland Ohio USA; ^3^ Department of Family Medicine Mountain Area Health Education Center Boone North Carolina USA; ^4^ University Program Case Western Reserve University School of Medicine Cleveland Ohio USA; ^5^ Department of Pediatrics, Morgan Stanley Children's Hospital Columbia University Irving Medical Center New York New York USA; ^6^ Environmental Health Watch Cleveland Ohio USA; ^7^ Office of Research and Evaluation McREL International Denver Colorado USA; ^8^ Office of Research and Evaluation Cleveland Metropolitan School District Cleveland Ohio USA; ^9^ Health and Opportunity Practice Group The Legal Aid Society of Cleveland Cleveland Ohio USA

**Keywords:** asthma, health equity, implementation science, school‐based health centers, translational research

## Abstract

**Background:**

There is a large body of research suggesting the role of school‐based health centers (SBHCs) in improving outcomes for children with asthma, but there are no evidence‐based guidelines for the care of children with asthma in SBHCs. We conducted a randomized trial to assess screening in children with asthma in an urban SBHC.

**Methods:**

Participants were screened for asthma triggers. The intervention group received home assessments and medical legal partnership (MLP) referrals as indicated. The primary outcome of asthma severity was assessed using the asthma control test (ACT). All participants completed semi‐structured interviews to evaluate their experience.

**Results:**

All families randomized to intervention qualified for and completed home remediation. There were no statistically significant differences in asthma severity. There was 100% retention of participants, and all participants rated their experience as good or excellent.

**Implications for School Health Policy, Practice and Equity:**

This study demonstrates a high prevalence of home‐based asthma triggers for children with asthma who receive care in SBHCs. Although not large enough to demonstrate significance in primary outcomes, participants were successfully recruited from a diverse population and retained through completion of the study. Participants rated their experience as good or excellent, suggesting that the recruitment and retention of diverse participants for clinical trials in SBHCs can be successful.

**Conclusions:**

Home‐based asthma triggers for children with asthma who receive care in SBHCs are prevalent. Although complex collaborations are required, SBHCs are a viable site for clinical trials. More research is needed to understand the benefit of interventions in SBHCs to reduce asthma severity.

## 1 Body

Asthma is a leading cause of chronic disease‐related school absenteeism [1], and large disparities in asthma‐related outcomes persist for children from minority, economic, and socially marginalized groups [2]. Although community resources exist, many families continue to experience unjust environmental exposures with limited access to primary care and other supports to manage asthma [3]. School‐Based Health Centers (SBHCs) offer an innovative approach to addressing the needs of children with asthma [4]. By reducing traditional barriers and coordinating with educational and community partners, SBHCs can play a role in improving access to services and resources [5]. Evidence suggesting improved health equity associated with SBHCs led to the Community Preventive Services Task Force (CPSTF) recommendation for the implementation of SBHCs [6]. However, the task force emphasized that further research was needed to understand optimal implementation. Although there is a large body of research suggesting the role of SBHCs in improving both health and educational outcomes, current evidence is limited by selection bias, maturational and historical effects, displacement effects, clustering effects, among other factors [7]. Since the CPSTF recommendation, there has been a dramatic rise in the number of SBHCs, with increases in governmental funding at both the national and state level [8]. Although the School‐Based Health Alliance (SBHA) has developed a set of core competencies for SBHC [9], there are no evidence‐based guidelines for SBHC implementation. Translational research that supports understanding the process of SBHC implementation is needed.

Primary care physicians are trained to screen children with asthma for triggers within the home environment. A significant body of evidence suggests home inspection and remediation for high‐risk children with asthma can improve asthma‐related outcomes [10]. However, evidence‐based standardized clinical screening tools to identify children with asthma who benefit from home assessment and remediation have not been established. Medical Legal Partnerships (MLPs) can improve housing conditions for pediatric patients [11], and home‐based remediation models have been found to improve asthma‐associated morbidity [12]. However, little is known about the utility of SBHCs as a referral source for home remediation or MLP referral.

Several research networks support capacity to develop evidence‐based guidelines for the care of patients in the primary care office setting, including the American Pediatrics Association's Pediatric Research in Office Settings and the National Institutes of Health's All of Us Framework. The Center for Disease Control's Adolescent and School Health, What Works in Schools Framework and Whole School, Whole Community, Whole Child Framework provide guidelines for implementation of public health initiatives in schools. The National Institutes of Health's National Center for Advancing Translational Sciences [13] was established to support implementation research to improve the health of patients and populations. However, no research networks exist to support the development of evidence‐based guidelines for implementation of primary care for children in the school setting.

We describe a small pilot randomized controlled trial (RCT) to understand the utilization of screening and referral for home‐based interventions and MLP for children with asthma in SBHCs.

The SBHC described in this study is a well‐established hospital‐sponsored Federally Qualified Health Center (FQHC) look‐alike multi‐site program that utilizes in‐school, telehealth, and mobile delivery to provide primary care, behavioral health, and dental services. Staffed with physicians, advance practice nurse practitioners, nurses, dentists, dental hygienists, medical and dental assistants, and community health workers, the SBHC provides services for children and school staff in an urban school district of around 36,000 students and an inner ring suburban district of around 25,000 students. The hospital sponsor is a nationally ranked non‐profit public health care system, primary affiliate of a local nationally ranked medical school, with a network of affiliated community‐based Patient Centered Medical Homes (PCMHs) and FQHC look‐alike centers. The SBHC acts as an extension service into the schools for the hospital system and associated PCMHs and FQHC look‐alike centers.

Environmental Health Watch (EHW) is Northeast Ohio's leading environmental justice organization. Formed in the early 1980's by concerned citizens and health professionals in response to pervasive health hazards in homes and communities, EHW has remained at the forefront of community‐led initiatives to address discriminatory inequality in housing, pollution, and toxic hazard exposure through environmentally conscious and sustainable solutions. EHW's foundational work in the development of the HHA contributed to the creation of the Principles of a Healthy Home used by the Department of Housing and Urban Development and the National Center for Healthy Housing.

The Legal Aid Society of Cleveland created the first MLP in Ohio, the fourth in the United States, when it formalized its program with the hospital sponsor in 2003. Legal Aid attorneys train physicians to recognize civil legal concerns impacting their patients. Physicians can then refer patients to Legal Aid to address illegal and discriminatory housing and neighborhood conditions, and ensure access to education, employment, food, and health assistance provided by law.

## 2 Methods

### 2.1 Participants

Participants ages 5–21 were eligible for inclusion if they were enrolled in the SBHC and had a diagnosis of asthma. Researchers identified eligible families from the SBHC clinical registry, which included all children enrolled in the SBHC.

At the time of the study, there were 4463 children in the registry, 51% female, 49% male, 29% Hispanic, 53% Black/African American, 19% White, 96% enrolled in Medicaid. Of children with a diagnosis of asthma, 46% were female, 54% male, 40% Hispanic, 52% Black/African American. The highest rates of asthma occurred in zip codes 44135, 44111, and 44102, where rates were 37%, 31%, and 29%, respectively. There were 726 children who met inclusion criteria. Recruitment began with children enrolled in two of the highest clinical volume elementary schools served by the SBHC and within closest proximity to a PCMH affiliated with the hospital sponsor.

### 2.2 Instrumentation

Primary outcome measure of asthma severity was assessed using the asthma control test (ACT) for participants aged 12 and over and the child asthma control test (cACT) for ages 11 and younger. Participants were screened for asthma triggers using the environmental screening tool (EST). The need for legal services was assessed using the community advocacy screening tool (CAST). The authors developed the EST and CAST, which consist of seven and three questions, respectively, and are completed by phone interview. The validity and reliability of the EST and CAST are unknown. Semi‐structured interview questions were developed using direct, indirect, probing, specifying, and interpreting structural elements to assess participants' experience in the study.

### 2.3 Procedure

**FIGURE 1:**
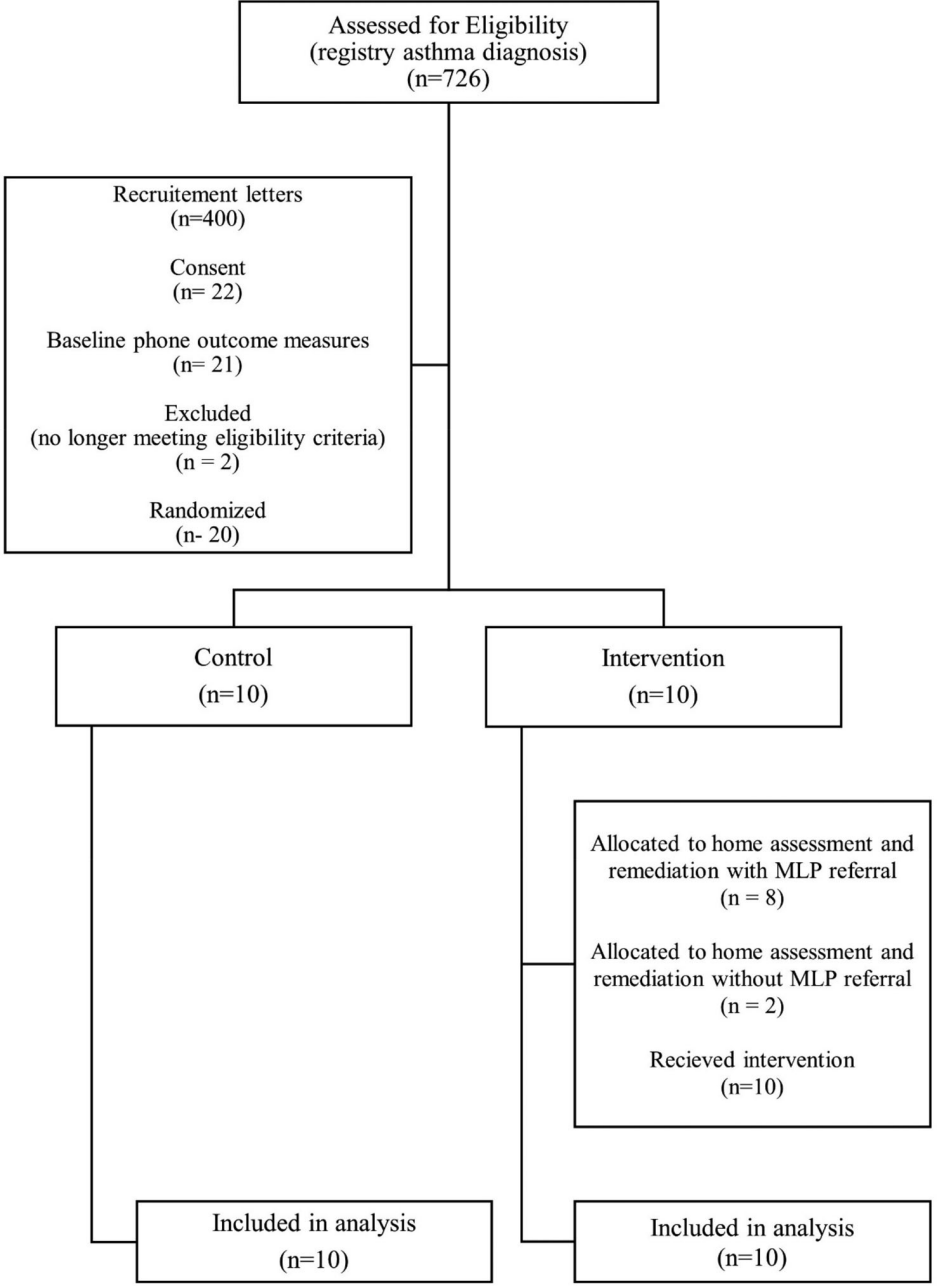
CONSORT flow diagram.

Families identified through the SBHC clinical registry were recruited through letters mailed to their homes followed by phone calls. Once enrolled, participants were screened for asthma triggers using the EST. The need for legal services was assessed using the CAST. Baseline outcome measures were assessed using the ACT and cACT. Researchers randomized participants into intervention and control groups using a random number generator for simple randomization with fixed allocation. Intervention participants who screened positive on the EST received a home assessment. Those who screened positive on the CAST received an MLP referral. The control group received no referrals. Home assessments were completed according to the healthy home audit (HHA) [14]. If indicated, home remediation was completed according to a predefined protocol [12]. Assessments by the MLP were completed as defined by the National Center for Medical Legal partnerships [15]. Based on the results of the EST, CAST, and HHA, participants were placed into intervention arms (Figure 1): home assessment and remediation with and without MLP referral. Semi‐structured interviews were completed with all families following 6‐month data collection.

### 2.4 Data Analysis

Study outcomes for intervention and control groups were analyzed using paired t tests. A three‐point difference in the ACT (12% difference) or a two‐point difference in cACT (10% difference) was considered a significant effect size. The mean and standard deviation of the ACT in this population are unknown but anticipated to be 20 and 4 based on prior studies [16]. Assuming a similar population, a sample size of 21 in the treatment group and 42 in the control group would provide a power of 80%, a type 1 (*α*) error of 5%, and a type II (*β*) error of 20% for a primary outcome of a three‐point difference in ACT.

HHAs were assessed for hazards identified, and remediation reports were assessed for supplies provided. Multiple authors independently analyzed semi‐structured interviews using inductive thematic analysis through familiarization, coding, generating themes, reviewing themes, and defining and naming themes.

## 3 Results

Of the 400 families who received recruitment letters and phone calls, 22 families were enrolled. Recruitment was anticipated to begin in January 2020 but was delayed until May of 2021 due to the COVID pandemic. Enrollment occurred between May and July of 2021, and final data was collected in April 2022. Two families were excluded from analysis due to no longer attending a school served by the SBHC and therefore no longer eligible for enrollment. Results reflect 20 families, 10 in the intervention group, 10 in the control group (Figure 1). Because one child in the intervention group split time between two homes, a total of 21 households were included. Participants were 70% male, 30% female, 65% Black/African American, 35% Hispanic (Table 1). Of the 21 households included in the analysis, 21 qualified for a home assessment and 16 qualified for a MLP referral.

**TABLE 1:** Participant demographics.

	Control	Intervention	Arm C1	Arm C2	Total	*p*
No. of participants	10	10	2	8	20	
Age, mean, (standard deviation)	11.3 (2.0)	10.2 (2.82)	10.5 (2.12)	10.13 (3.09)	10.75 (2.45)	0.33
Female, no. (%)	4 (40%)	2 (20%)	0 (0%)	2 (25%)	14 (70%)	0.33
Male, no. (%)	6 (60%)	8 (80%)	2 (100%)	6 (75%)	6 (30%)	
Race, no. (%)						
Black or African American	6 (60%)	7 (70%)	1 (50%)	6 (75%)	13 (65%)	0.49
White	1 (10%)	1 (10%)	1 (50%)	1 (13%)	3 (15%)	
Ethnicity, no. (%)						0.64
Hispanic	4 (40%)	3 (30%)	1 (50%)	2 (25%)	7 (35%)	
Non‐Hispanic	6 (60%)	7 (70%)	1 (50%)	6 (75%)	13 (65%)	

**FIGURE 2:**
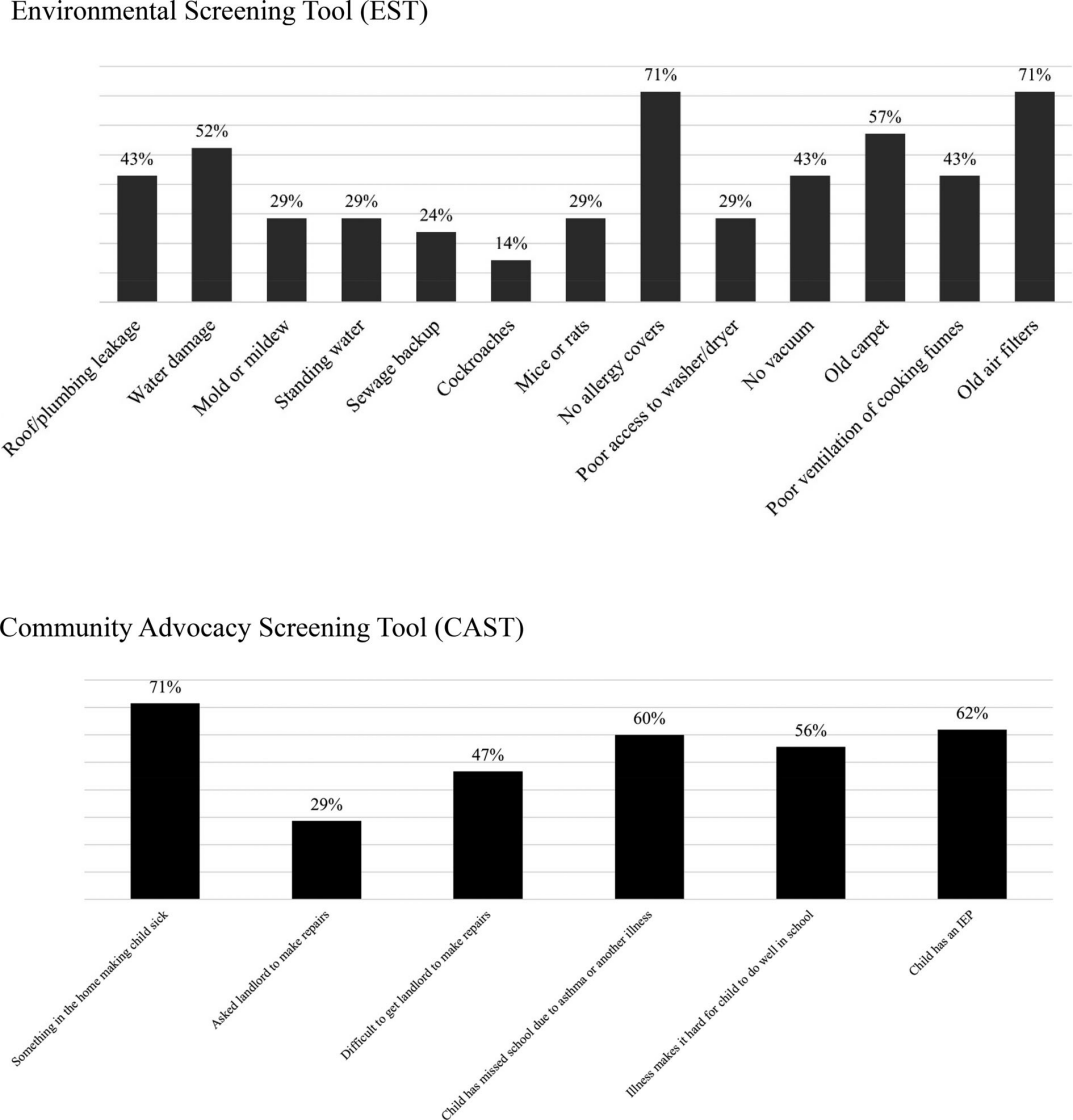
Screening results. (a) Environmental screening tool (EST), (b) community advocacy screening tool (CAST).

Results of the EST and CAST are shown in Figure 2. The most cited asthma triggers included forced air systems with old air filters, lack of pillow and mattress covers and old carpet, and water damage. Families were majority renters: 71% rented and 29% owned their home. A large majority of families (71%) endorsed concern regarding home conditions affecting their child's health. A majority (60%) of families reported their child had missed school due to asthma or another illness, and 56% reported asthma or another illness made it difficult for their child to do well in school.

All families randomized to intervention completed a home assessment and remediation. The number of identified hazards ranged from 3 to 7, with an average of 5 hazards per household. The number of supplies ranged from 3 to 14, with an average of 7. The cost per family for repairs and supplies ranged between $356 and $1072, with an average cost of $754.45. The supply most commonly noted to be helpful was a vacuum cleaner. Other reported helpful supplies included air purifiers, totes, humidifiers, and allergy covers. No adverse events were reported.

**FIGURE 3:**
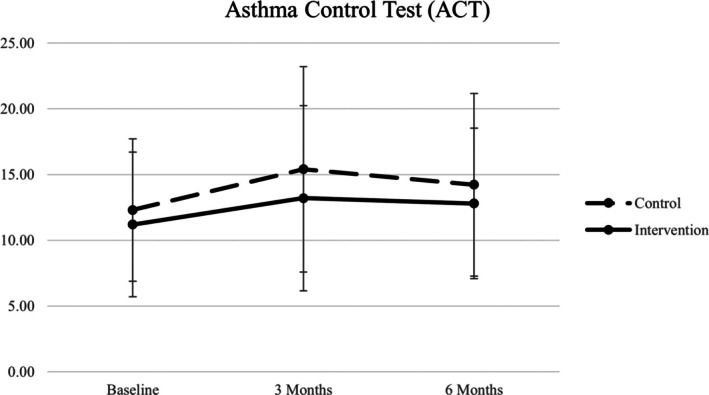
Asthma control test (ACT) results.

Results of the primary outcome measure at baseline, 3, and 6 months are shown in Figure 3. There was no statistically significant difference in asthma control between the intervention and control groups.

Semi‐structured interview results are shown in Table 2. All participants rated their experience as good or excellent. For participants in the intervention group, 100% reported that the family benefited from being in the study, from knowing what to look for in the home that could make asthma worse, better asthma control and/or cleaner home/air. Of all participants, 55% said they participated in the study to advance science or help others with asthma.

**TABLE 2:** Participant semi structured interview results.

First time in research study? Yes 20 (100%)					
Why do the study?	Help me/my family/my child better understand, control asthma 14 (70%)	Advance science, help others with asthma 11 (55%)	SCBH suggested it 1 (5%)	You called and asked 1 (5%)	
Anything you want to share about being a part of the study?	Researchers were flexible, understanding, tailored to our time/availability 7 (35%)	Liked phone calls, ease of communication, frequent check ins 7 (35%)	Liked helping others 3 (15%)	Learned a lot 3 (15%)	Positive experience 5 (25%)
Anything you want to share about how the study impacted other children and/or family members in your home?		Family benefitted, knew what to look for in the home that could be making asthma worse, better asthma control and/or cleaner home/air 10 (100%)		Improved medication adherence for child and/or other family 1 (10%)	
Anything you want to share about how study has impacted your child's education?		Better sleep 3 (30%)	Better insight into asthma control/improved ability to use medication and/or advocate for self 5 (50%)	Missed less school/did better in school 2 (20%)	
Home assessment experience? Suggestions to improve scheduling or otherwise?		Positive experience 4 (40%)	Technicians were flexible, tailored to our time/availability 5 (50%)	Technicians were kind, welcoming, professional, helpful 3 (30%)	
Supplies most helpful	Clothing and bedding totes/food and refuse containers 3 (30%)	Pillow/mattress protectors 3 (30%)	Air purifier 5 (50%)	Dehumidifier 2 (20%)	Vacuum 8 (80%)
Supplies you wish you had received	Additional accessories for supplies (filters for air conditioner, hose for dehumidifier) 2 (20%)		Bagless vacuum 1 (10%)	Door sealants/thresholds 2 (20%)	
MLP experience suggestions?	Didn't think it would be helpful 3 (30%)	Already had an open case 1 (10%)		Too busy 1 (10%)	
MLP helpful? Why/why not?			Helpful with improving IEP services 1 (10%)		
Overall experience with study		Bad 0 (0%)	Average 0 (0%)	Good 4 (40%)	Excellent 6 (60%)

## 4 Discussion

Of the many challenges to SBHC implementation, the requirement for collaboration between health and education systems, with vastly different goals and priorities, poses several challenges [17]. However, there is growing recognition among educational systems that children cannot learn if they are not healthy, and among healthcare systems that value‐based contracts can be more successful with the engagement of high‐risk populations. With the expansion of Medicaid value‐based payments [18] and newer evidence‐based clinical recommendations showing promise in reducing asthma‐associated morbidity [19], caring for children with asthma, the leading cause of chronic disease‐related school absenteeism, within schools can provide a unique opportunity to collaborate. However, evidence‐based guidelines to optimize asthma care within SBHCs are nonexistent.

Generating the evidence to support evidence‐based guidelines for SBHC implementation, including clinical trials of interventions aimed at improving both health and educational outcomes, poses significant challenges. Trials require a high level of institutional support, expertise, and collaboration to obtain data use agreements (DUAs) and memorandums of understanding (MOUs) from cross‐sector educational, healthcare, and community partners that ensure adherence to required Family Educational Rights and Privacy Act (FERPA) and the Health Insurance Portability and Accountability Act (HIPAA) regulations. SBHC success often depends on multi‐organizational cross‐sector collaboration. However, budgetary support and resource allocation across sectors as well as ensuring all educational and community partners are supported through requirements of hospital Institutional Research Board (IRB) protocols and approval with long‐term maintenance of protocols can pose additional challenges. Successful completion of any clinical trial requires all collaborators to support an extended commitment, including staff retention and/or adequate succession planning. Within cross‐sector collaborations, particularly with public entities such as schools or when leadership and strategic planning are highly dynamic, shifting priorities can make long‐term research planning particularly challenging.

We describe a small pilot RCT in an urban SBHC during the 2021–2022 school year to assess the use of screening tools in SBHC for the care of children with asthma. Although we benefitted significantly from the expertise of an academic center with extensive experience in community‐based research, we faced several challenges in ensuring appropriate DUAs and MOUs across partners. We were fortunate to have educational and community‐based environmental advocacy institutions, both with prior research experience, willing to engage extensively with the hospital‐based IRB to develop protocols and obtain DUAs with adequate privacy protections. This allowed for optimal communication and coordination between research staff and community partners, which may have contributed to 100% retention of families throughout the study. Although the hospital system has a well‐established MLP with its own MOU, that agreement did not support protections for direct data sharing between research staff and MLP colleagues, leading to restricted direct communication and coordination with the MLP. This may have contributed to low numbers of participants fully utilizing MLP resources. Based on this experience, we would recommend any clinical trial in an SBHC of a proposed intervention aimed at improving both health and educational outcomes ensure foundational structural support for robust and long‐term planning regarding optimal DUAs and MOUs, preferably with all educational and community partners playing a central role in data use and protections.

Our results of the EST identified home‐based triggers among participants that were common. Home assessment and remediation findings correlated closely with screening, suggesting that screening can accurately identify children who may benefit from home assessment. CAST results identified a large percentage of participants who were concerned that something in the home was making their child sick, and a significant percentage of participants reported their child had missed school due to asthma and that asthma or another illness made it difficult for their child to do well in school. This suggests that screening can identify children who would benefit from an MLP to assist families with establishing adequate educational supports, including Individualized Education Plans or 504 plans under the Americans with Disabilities Act.

Semi‐structured interview results identified that all participants rated their experience as good or excellent. All participants in the intervention group reported a benefit from being in the study, and a large percentage reported they participated in the study to advance science or help others with asthma. This suggests that recruitment and retention for research trials in SBHCs can be successful.

## 5 Implication for School Health Policy and Practice

Situated at the intersection of public health and clinical medicine, SBHCs address the needs of individual patients and the community. Professionals providing healthcare services in schools collaborate across sectors with a shared goal of improving both health and educational outcomes in the populations they serve. Providing healthcare services in schools requires not only foundational expertise in clinical and preventive medicine but also in both the healthcare system and educational system, systems that continue to grow in capacity and complexity within continuously evolving regulatory and compliance environments. As investment in SBHCs grows, generating the evidence to support evidence‐based guidelines for implementation, including clinical trials of interventions aimed at improving both health and educational outcomes, is necessary. Establishing centers of translational research in SBHCs may be one way to support the research needed to develop evidence‐based guidelines for SBHC implementation.

## 6 Limitations

Our study has several limitations, most notably low sample size and subsequent inadequate power to identify significant differences in primary outcome between intervention and control. Recruitment was constrained by the COVID pandemic as well as English‐only research staff and recruitment materials that restricted our capacity to enroll non‐English speaking participants. We also experienced several factors limiting recruitment similar to previously reported factors in recruitment of minority populations [20], including competing demands, health insurance coverage, and legal status concerns as well as transient housing and communication access. However, we were able to realize 100% retention of families throughout the study, possibly attributable to high engagement from community‐based research staff that interacted on a regular basis with participant families. Even with significant recruitment barriers, we were able to recruit and retain a diverse sample of participants.

## 7 Conclusions

We describe a small pilot RCT in an urban SBHC during the 2021–2022 school year to assess the use of screening tools in SBHC for the care of children with asthma. This study documents a high prevalence of home‐based asthma triggers for children with asthma who receive care in SBHCs. Although not large enough to demonstrate statistical significance in primary outcomes, our study successfully recruited from a diverse population and retained all participants through completion of the study. Participants reported a benefit from participation, and all rated their experience as good or excellent, suggesting that recruitment and retention for research trials in SBHCs can be successful.

Evidence‐based guidelines for SBHC interventions aimed at improving both health and educational outcomes are nonexistent. More clinical trials to generate evidence to develop guidelines are necessary to ensure quality and effectiveness of care during the expansion and implementation of SBHCs. Although multiple partner collaboration is complex, our study suggests SBHCs are a viable site for clinical trials. More research is needed to understand if screening in SBHCs can reduce asthma severity in children with asthma.

## Ethics Statement

This study was approved by the Institutional Review Board of the MetroHealth Medical System Protocol ID: IRB18‐00771.

## Conflicts of Interest

The authors declare no conflicts of interest.
